# Hereditary transthyretine amyloidosis in Slovenia

**DOI:** 10.1186/1750-1172-10-S1-P17

**Published:** 2015-11-02

**Authors:** Janez Zidar

**Affiliations:** 1 University Medical Centre Ljubljana, Institute of Clinical Neurophysiology, 1525, Ljubljana, Slovenia

## 

The prevalence of transthyretin (TTR) amyloidosis in Europe is supposed to be less than one per 100,000 individuals. Slovenia is a rather small country with approximately two million inhabitants and we know of two families with 4 patients with this disease. The first patient (family A) was diagnosed in 2008 when also one of the asymptomatic family members who agreed to molecular genetic testing was found to be a carrier. Other family members up to now disagreed with clinical, laboratory and molecular genetic testing. Patients from family B were diagnosed in 2014. Their siblings were found not to be carriers.

The index case from family A was diagnosed at the age of 54. The TTR gene mutation was characterised as Val122Ala. First symptoms were due to dysautonomia and appeared 4 years earlier in the form of abdominal colic associated with vomiting and orthostatic hypotension. He also had symptoms and signs of length-dependent axonal polyneuropathy and hypertrophic restrictive cardiomyopathy. He died the day after liver transplantation.

The disease of the family's B index case started approximately one and a half year before the diagnosis with pain in his feet, orthostatic hypotension, sexual impotence, diarrhoea alternating with constipation, and weight loss. Later on he also noticed weakness in his lower limbs but is still able to walk unaided. The Val30Ala mutation was found. Except to polyneuropathy no other organs are affected. He is treated with tafamidis. His sister is asymptomatic but is having electrophysiological signs of polyneuropathy and cardiac vagal denervation. She recently also started taking tafamidis.

With increasing awareness of the transthyretin amyloidosis, especially amongst neurologists and cardiologists in our country, we expect to find some more patients in the near future, particularly among members of the affected families. Two other unrelated patients are currently undergoing testing, one of them with transthyretin amyloid tissue deposits.

